# Supplementary material for: Diabetes and physical activity: A prospective cohort study
Source: PLoS One. 2022 Oct 26;17(10):e0276761. doi: 10.1371/journal.pone.0276761 (PMC9604951; doi:10.1371/journal.pone.0276761)
Supplement: S1 Appendix — (DOCX) [file pone.0276761.s001.docx]

**Questions used in calculating the MET score**

The following is an excerpt from the HABITAT questionnaire.


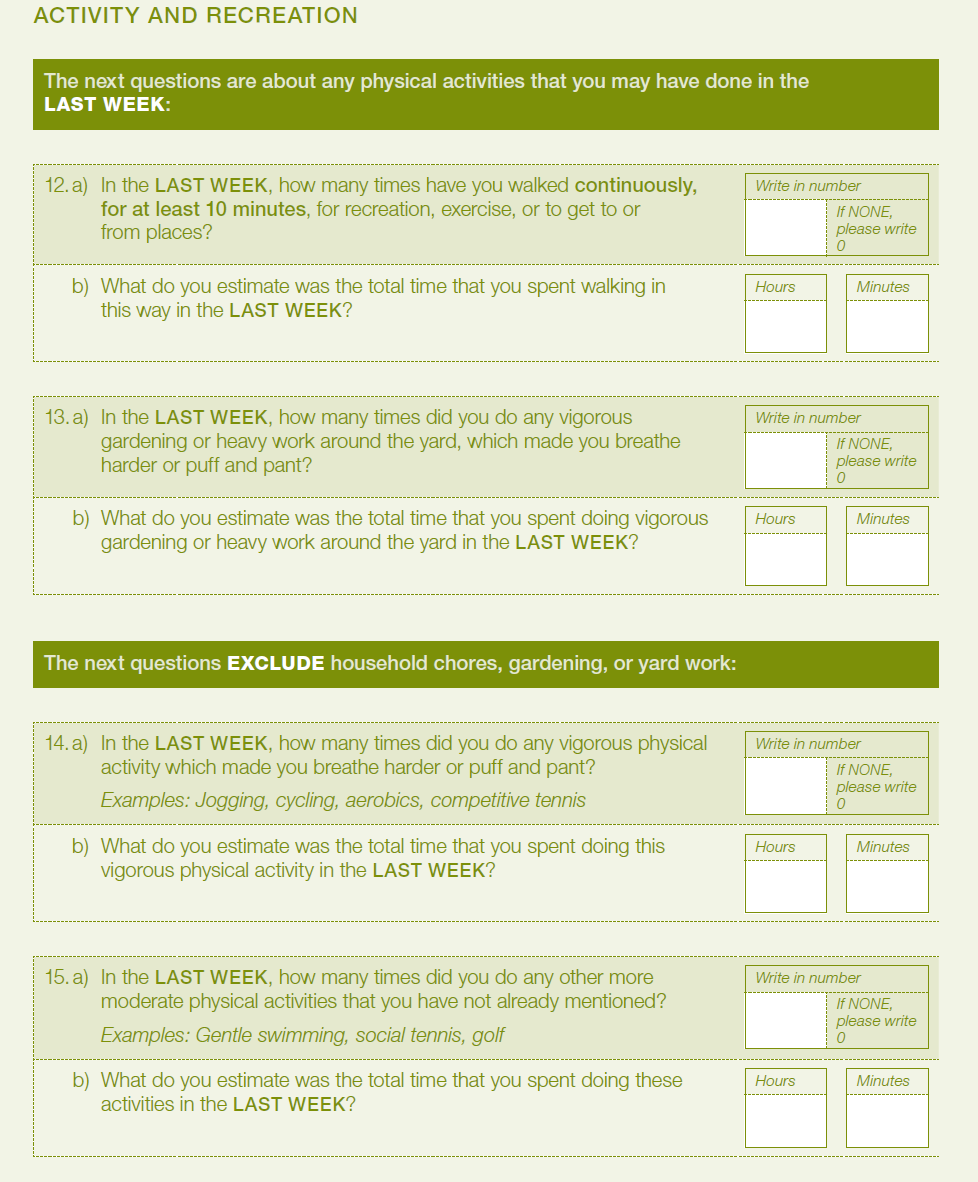


**The RE-EM tree model**

Formulation of the RE-EM tree model is:

$$y_{it}= Z_{it}\mathbf{b}_{i}+f\left( X_{it1}, . ..,X_{itk} \right)+ \varepsilon_{it}$$

$\binom{\begin{aligned} \varepsilon_{i1} \\ . \\ . \\ . \end{aligned}}{\varepsilon_{{iT}_{i}}}\sim\mathrm{Normal}(\boldsymbol{0},R_{i})$ and $\mathbf{b}_{i}\sim\mathrm{Normal}(\boldsymbol{0},D)$

where (*i,t*) refers to a panel of individuals *i* = 1,...,*I* at times *t* = 1,...,$T_{i}$. Each individual is associated with multiple observations, each of which has a vector of covariates, $\mathbf{X}_{\mathbf{it}}$ = $\left( X_{it1}, . ..,X_{itk} \right)^{T}$ and an outcome, $y_{it}$. The attributes may be constant over time, constant across individuals, or varying across time and individuals. The design matrix, $Z_{it}$, accounts for the differences between individuals across time periods. $\mathbf{b}_{i}$ accounts for a vector of unknown time-constant individual-specific effects. The errors, $\varepsilon_{it}$, are assumed to be independent across individuals and are uncorrelated with the effects, $\mathbf{b}_{i}$. *f* is a function that is linear in the parameters and the **b_i_** are taken as fixed or potentially correlated with the attributes which makes *f* a linear fixed effect.

The steps of the Unbiased RE-EM tree algorithm [1] are:

1. Initialize ${\hat{\mathbf{b}}}_{i}$(estimated random effects) to zero.
2. Iterate through the steps below until ${\hat{\mathbf{b}}}_{i}$ converge (based on change in likelihood or restricted likelihood function values being less than a pre-defined tolerance value):
3. Estimate a regression tree approximating the population-level effects *f,* based on the target variable $y_{it}- Z_{it}{\hat{\mathbf{b}}}_{i}$and covariates, $\boldsymbol{X}_{it}=(X_{it1},\ldots,X_{itk})$, for individuals *i* = 1,…..,*I* at times t = 1,….,$T_{i}$. Use this regression tree to create a set of indicator variables, *I(*$x_{it}\epsilon g_{p}),$ where $g_{p}$ ranges over all terminal nodes in the tree.
4. Fit the linear mixed effect model,

$y_{it}= Z_{it}\mathbf{b}_{i}+ \sum_{p} I (X_{it} \epsilon g_{p})\mu_{p}+ \varepsilon_{it}$

and extract ${\hat{\mathbf{b}}}_{i}$ from the estimated model.

1. Replace the predicted response at each terminal node of the tree with the estimated population level predicted response $\hat{\mu}_{p}$from the linear mixed effects model fit in 2b. Reject the null hypothesis when the minimum of the adjusted P-values is less than a prespeciﬁed nominal level α and otherwise stop the algorithm.

References

1. Fu W, Simonoff JS. Unbiased regression trees for longitudinal and clustered data. Computational Statistics & Data Analysis. 2015;88:53-74.
