# Supplementary material for: Diabetes and physical activity: A prospective cohort study
Source: PLoS One. 2022 Oct 26;17(10):e0276761. doi: 10.1371/journal.pone.0276761 (PMC9604951; doi:10.1371/journal.pone.0276761)
Supplement: S1 Table — (PDF) [file pone.0276761.s002.pdf]

**Variable**	***Affects***									
	PA	***Diabetes***	***Age***	***Sleep***	***Sex***	***Diet***	***BMI***	***Education***	***Depression***	***Neig'hood***
**PA**				[1]			[2, 3]		[4]	
**Age**	[5]	[6]		[7]			[8, 9]			
**Sleep**		[10]				[11, 12]	[10]			
**Sex**		[13]				[14]				
**Diet**		[15]					[16]			
**BMI**		[15]								
**Education**	[17]					[17]				
**Depression**				[18]		[19]				
**Neig'hood**	[20]			[21, 22]		[23, 24]			[25, 26]	

References

1. Uchida S, Shioda K, Morita Y, Kubota C, Ganeko M, Takeda N. Exercise Effects on Sleep Physiology. Frontiers in Neurology. 2012;3:48.
2. Swift DL, Johannsen NM, Lavie CJ, Earnest CP, Church TS. The role of exercise and physical activity in weight loss and maintenance. Progress in cardiovascular diseases. 2014;56(4):441-447.
3. Jakicic JM. The Role of Physical Activity in Prevention and Treatment of Body Weight Gain in Adults. The Journal of Nutrition. 2002;132(12):3826S-3829S.
4. Schuch FB, Vancampfort D, Richards J, Rosenbaum S, Ward PB, Stubbs B. Exercise as a treatment for depression: A meta-analysis adjusting for publication bias. Journal of Psychiatric Research. 2016;77:42-51.
5. McPhee JS, French DP, Jackson D, Nazroo J, Pendleton N, Degens H. Physical activity in older age: perspectives for healthy ageing and frailty. Biogerontology. 2016;17(3):567-580.
6. Kirkman MS, Briscoe VJ, Clark N, Florez H, Haas LB, Halter JB, et al. Diabetes in Older Adults. Diabetes Care. 2012;35(12):2650-2664.
7. Suzuki K, Miyamoto M, Hirata K. Sleep disorders in the elderly: Diagnosis and management. Journal of general and family medicine. 2017;18(2):61-71.
8. Kuczmarski RJ, Flegal KM, Campbell SM, Johnson CL. Increasing Prevalence of Overweight Among US Adults: The National Health and Nutrition Examination Surveys, 1960 to 1991. JAMA. 1994;272(3):205-211.
9. Roberts SB, Williamson DF. Causes of Adult Weight Gain. The Journal of Nutrition. 2002;132(12):3824S-3825S.
10. Knutson KL, Van Cauter E. Associations between sleep loss and increased risk of obesity and diabetes. Annals of the New York Academy of Sciences. 2008;1129:287-304.
11. Chaput J-P. Sleep patterns, diet quality and energy balance. Physiology & Behavior. 2014;134:86-91.
12. Leproult R, Van Cauter E. Role of sleep and sleep loss in hormonal release and metabolism. Endocrine development. 2010;17:11-21.
13. De Paoli M, Werstuck GH. Role of Estrogen in Type 1 and Type 2 Diabetes Mellitus: A Review of Clinical and Preclinical Data. Canadian Journal of Diabetes. 2020.
14. Bennett E, Peters SAE, Woodward M. Sex differences in macronutrient intake and adherence to dietary recommendations: findings from the UK Biobank. BMJ Open. 2018;8(4):e020017.
15. Forouhi NG, Misra A, Mohan V, Taylor R, Yancy W. Dietary and nutritional approaches for prevention and management of type 2 diabetes. BMJ. 2018;361:k2234.
16. Duvigneaud N, Wijndaele K, Matton L, Philippaerts R, Lefevre J, Thomis M, et al. Dietary factors associated with obesity indicators and level of sports participation in Flemish adults: a cross-sectional study. Nutrition journal. 2007;6:26-26.
17. Pampel FC, Krueger PM, Denney JT. Socioeconomic Disparities in Health Behaviors. Annual review of sociology. 2010;36:349-370.
18. Lauer CJ, Wiegand M, Krieg J-C. All-night electroencephalographic sleep and cranial computed tomography in depression. European Archives of Psychiatry and Clinical Neuroscience. 1992;242(2):59-68.

19. Appelhans BM, Whited MC, Schneider KL, Ma Y, Oleski JL, Merriam PA, et al. Depression Severity, Diet Quality, and Physical Activity in Women with Obesity and Depression. Journal of the Academy of Nutrition and Dietetics. 2012;112(5):693-698.
20. Li F, Fisher KJ, Brownson RC, Bosworth M. Multilevel modelling of built environment characteristics related to neighbourhood walking activity in older adults. Journal of Epidemiology and Community Health. 2005;59(7):558-564.
21. Hale L, Hill TD, Friedman E, Nieto FJ, Galvao LW, Engelman CD, et al. Perceived neighborhood quality, sleep quality, and health status: evidence from the Survey of the Health of Wisconsin. Social science & medicine (1982). 2013;79:16-22.
22. Hill TD, Trinh HN, Wen M, Hale L. Perceived neighborhood safety and sleep quality: a global analysis of six countries. Sleep Medicine. 2016;18:56-60.
23. Black C, Moon G, Baird J. Dietary inequalities: What is the evidence for the effect of the neighbourhood food environment? Health & Place. 2014;27:229-242.
24. Bodor JN, Rose D, Farley TA, Swalm C, Scott SK. Neighbourhood fruit and vegetable availability and consumption: the role of small food stores in an urban environment. Public Health Nutrition. 2008;11(4):413-420.
25. Mair C, Roux AVD, Galea S. Are neighbourhood characteristics associated with depressive symptoms? A review of evidence. Journal of Epidemiology and Community Health. 2008;62(11):940-946.
26. O′Campo P, Wheaton B, Nisenbaum R, Glazier RH, Dunn JR, Chambers C. The Neighbourhood Effects on Health and Well-being (NEHW) study. Health & Place. 2015;31:65-74.
