# Supplementary figures and images for: Diabetes and physical activity: A prospective cohort study
Source: PLoS One. 2022 Oct 26;17(10):e0276761. doi: 10.1371/journal.pone.0276761 (PMC9604951; doi:10.1371/journal.pone.0276761)

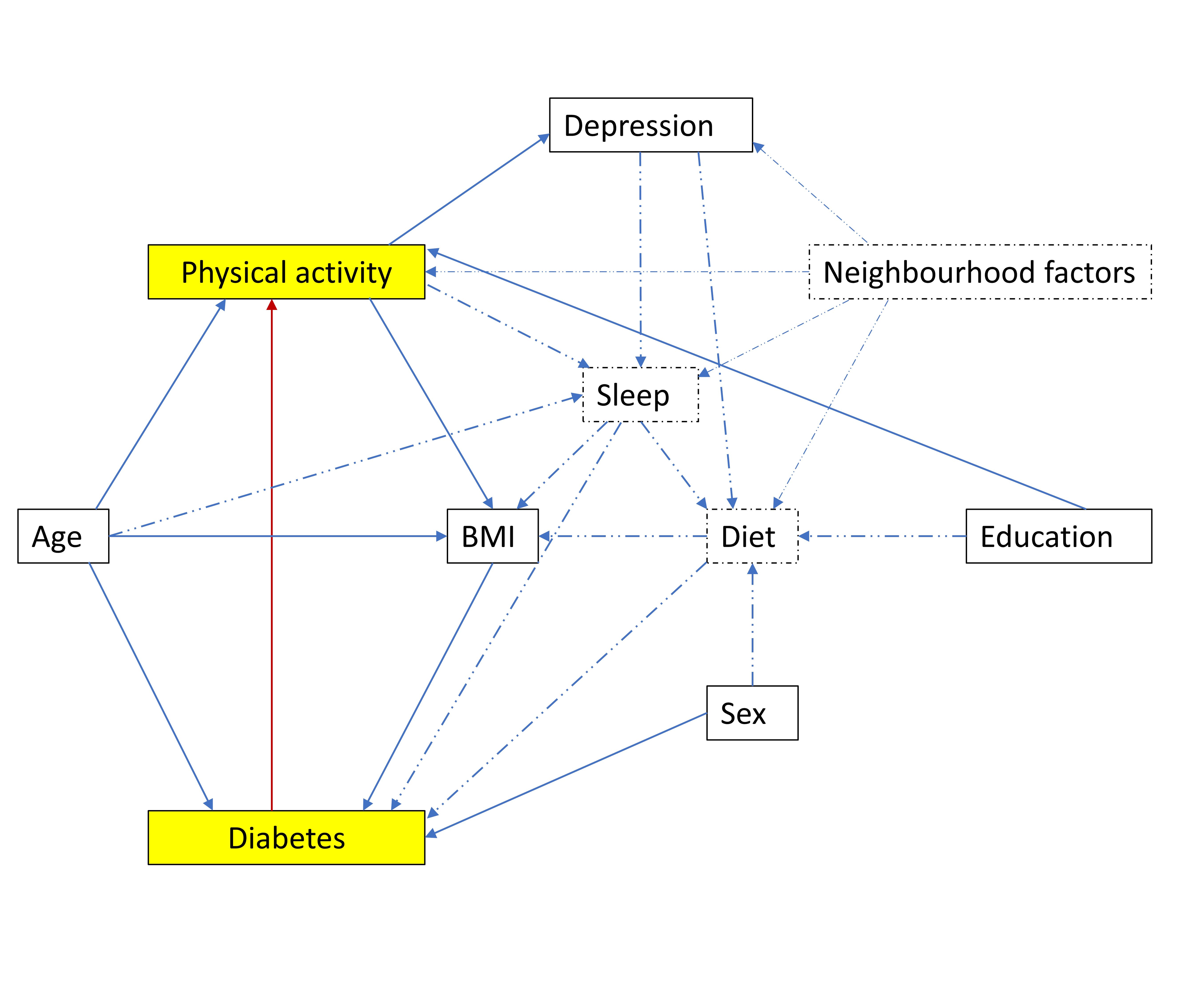

Supplement: S1 Fig — Notes: Dashed boxes (with associated dashed arrows) indicate the variable was not included in the analysis. See S1 Table for the evidence underlying these relationships. (TIF) [file pone.0276761.s003.tif]

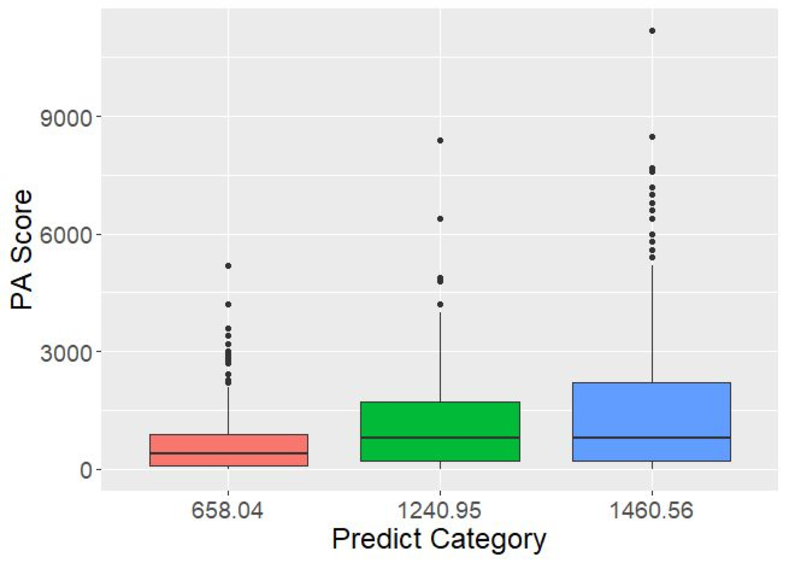

Supplement: S2 Fig — (TIF) [file pone.0276761.s004.tif]

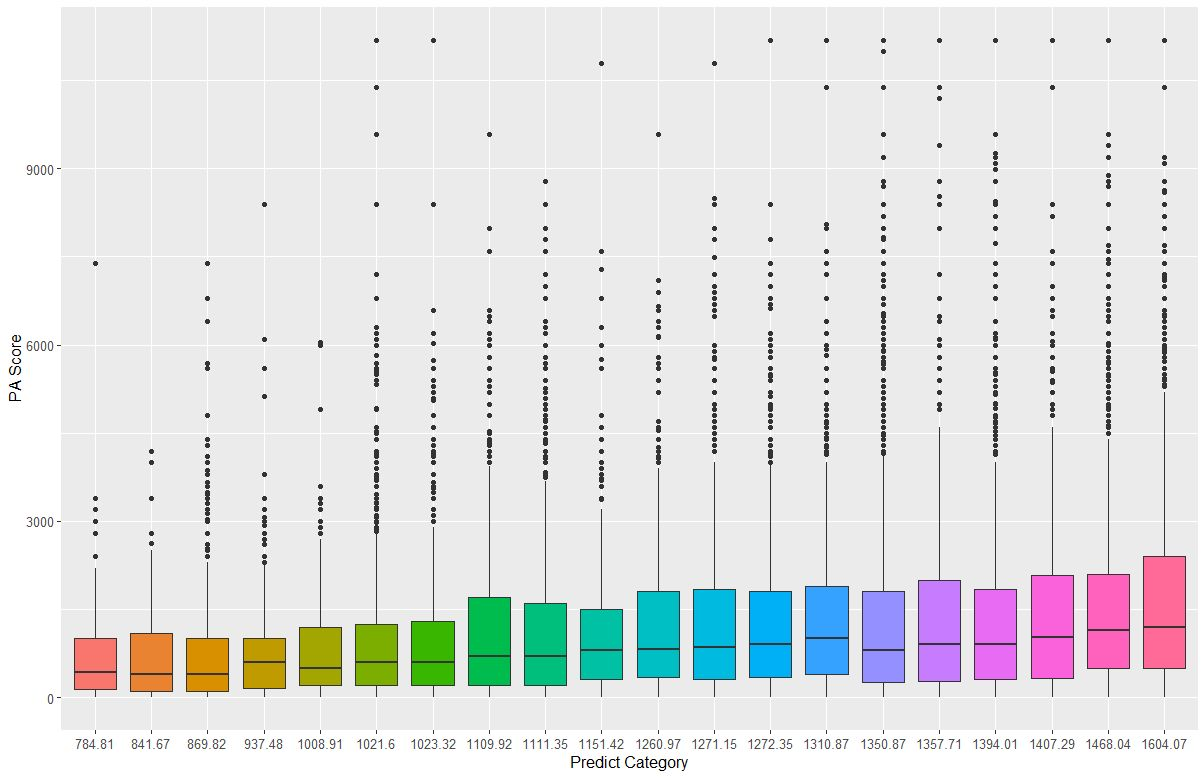

Supplement: S3 Fig — (TIF) [file pone.0276761.s005.tif]

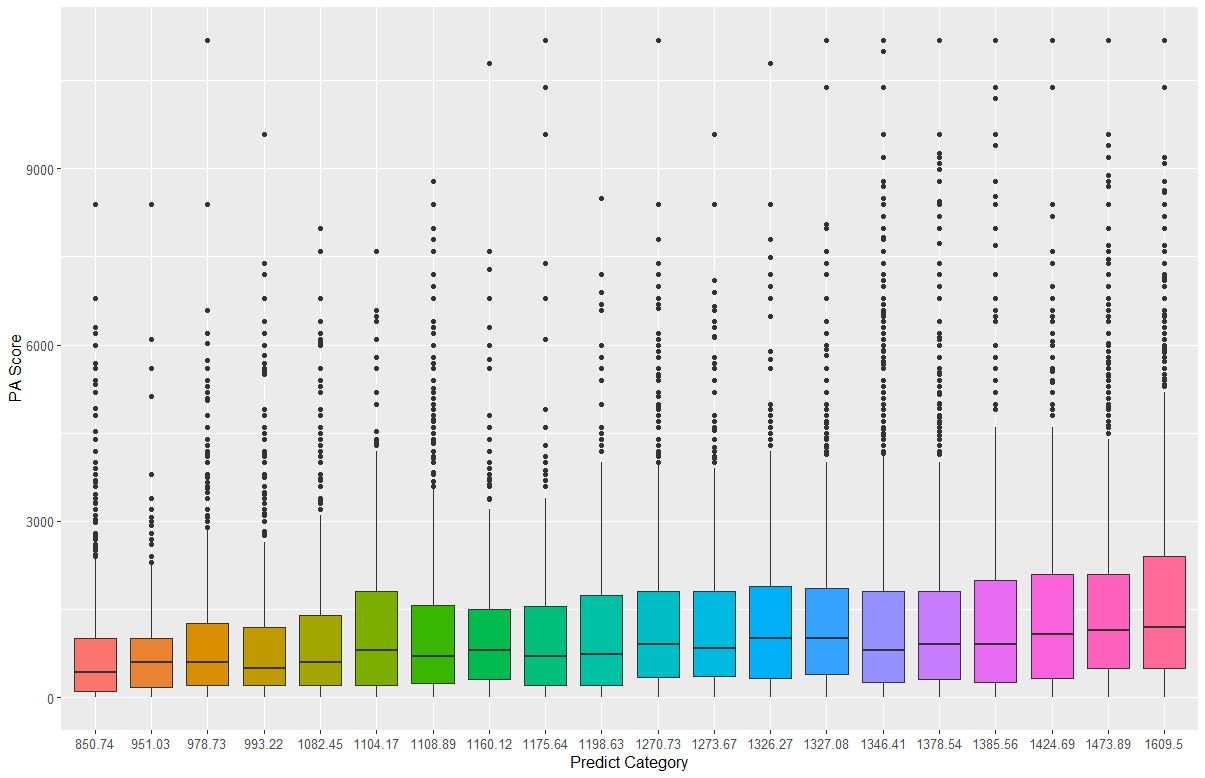

Supplement: S4 Fig — (TIF) [file pone.0276761.s006.tif]
