# Supplementary material for: Diabetes and physical activity: A prospective cohort study
Source: PLoS One. 2022 Oct 26;17(10):e0276761. doi: 10.1371/journal.pone.0276761 (PMC9604951; doi:10.1371/journal.pone.0276761)
Supplement: Attachment — Submitted filename: PLOS Response to reviewers R2.pdf [file pone.0276761.s007.pdf]

RESPONSE TO STATISTICAL REVIEWER'S FEEDBACK

1- Abstract: Provide p-values to support the following statement. "At study entry, those with diabetes had a higher median age of 58 years and a lower median physical activity of 699 MET.min/week than people without diabetes (53 years and 849 MET.min/week)."

Response: We agree that it is important to understand if these differences are likely to be genuine, and have modified the abstract to now include 95% CIs for each of these terms, as follows:

At study entry, those with diabetes had a higher median age of 58 years (95% CI: 57 - 60) and a lower median physical activity of 699 MET.min/week (95% CI: 599 - 799) than people without diabetes (53 years (95% CI: 53 - 53) and 849 MET.min/week (95% CI: 799 - 899)).

While p-values would be more concise, their general unreliability and confusion around interpretation (e.g. highlighted in https://amstat.tandfonline.com/doi/full/10.1080/00031305.2016.1154108#.YzPZ_3ZBwuU) means we prefer to report confidence intervals.

2- Lines 253-262: Provide measures of dispersion of the median ages and physical activity scores.

Response: This has been altered as follows:

The median age of all respondents in 2009 was 53 years (95% CI:53 – 54), and the median physical activity score was 833 MET.min/week (95% CI:799 – 899)…. The 362 respondents with diabetes had a higher median age of 58 years (95% CI: 57 - 60) and a lower median physical activity of 699 MET.min/week (95% CI: 599 - 799) than people without diabetes (53 years (95% CI: 53 - 53) and 849 MET.min/week (95% CI: 799 - 899), respectively).

3- The standard statistical term for average is mean.

Response: We have now altered average to mean in lines 264-265, plus inserted "(mean)" beside average in Figure 2 footnote. While the manuscript text often mentions average in the results, the aim is to make it more accessible to readers who may be less comfortable with statistical terminology.

Lines 264 to 265 now read as:
At wave 2, people with diabetes had a higher physical activity score (mean: 1343; median 716 MET.min/week) than at wave 5 (mean: 1131; median 599).

4- Table 1: Typically missing categories are presented last in each listing, and they are not included in the percentage calculation.

Response: We have moved the missing categories to be last, but still include them in the percent calculations since this is important information for readers, and many are so small they have a negligible impact on the other percentages. We have also added a "0 (0%)" for those with diabetes missing gender.

The new Table 1 is shown below.

Table 1. Characteristics of the analytic study sample at entry, by diabetes status.

	Respondents N (%)		Physical Activity† Median (Inter-Quartile Range)	
	**Diabetes**	**Non-Diabetes**	**Diabetes**	**Non-Diabetes**
**Total**	362 (5%)	7338 (95%)	699 (133 - 1698)	849 (300 - 1898)
**Gender**				
Men	186 (51%)	3096 (42%)	899 (200 - 2398)	999 (316 - 1998)
Women	176 (49%)	4241 (58%)	500 (100 - 1199)	799 (300 - 1764)
Missing	0 (0%)	1 (0.01%)		
**Education**				
No Post School Qualification	163 (45%)	2667 (36%)	399 (100 - 1199)	699 (200 - 1598)
Vocational Qualification	64 (18%)	1290 (18%)	799 (258 - 1723)	899 (300 - 1998)
Diploma	41 (11%)	858 (12%)	1299 (300 - 2398)	899 (400 - 1998)
Bachelor Degree or Higher	94 (26%)	2507 (34%)	999 (325 - 2473)	999 (400 - 1998)
Missing	0 (0%)	16 (0.2%)		416 (92 - 1232)
**BMI**				
Underweight	1 (0.2%)	104 (1%)		599 (200 - 1824)
Normal Weight	53 (15%)	2831 (39%)	799 (300 - 2398)	999 (400 - 1998)
Overweight	115 (32%)	2858 (39%)	899 (250 - 1998)	899 (300 - 1948)
Class 1 Obesity	109 (30%)	1082 (15%)	599 (100 - 1399)	666 (200 - 1598)
Class 2 Obesity	40 (11%)	228 (3%)	483 (125 - 1698)	400 (100 - 1099)
Class 3 Obesity	37 (10%)	125 (2%)	500 (0 - 1199)	400 (67 - 1199)
Missing	7 (2%)	110 (1%)	599 (225 - 1049)	599 (200 - 1099)
**Psychological Distress**				
Low/No Distress	204 (56%)	4835 (66%)	799 (200 - 1998)	899 (333 - 1998)
Moderate Distress	85 (24%)	1606 (22%)	599 (100 - 1399)	799 (266 - 1698)
High Distress	40 (11%)	499 (7%)	549 (100 - 1474)	699 (200 - 1598)
Very High Distress	21 (6%)	270 (3%)	400 (100 - 1199)	599 (100 - 1465)
Missing	12 (3%)	128 (2%)	553 (29 - 2198)	1024 (300 - 2023)
**Annual Gross Household Income (AUD)**				
< $25,999	73 (20%)	638 (9%)	500 (67 - 1598)	799 (200 - 1690)
$26,000 - 51,999	79 (22%)	1234 (17%)	799 (200 - 1948)	699 (200 - 1698)
$52,000 - 72,799	50 (14%)	968 (13%)	599 (108 - 1399)	799 (266 - 1798)
$72,800 - 129,999	67 (18%)	1924 (26%)	749 (200 - 1948)	833 (300 - 1798)
≥ $130,000	39 (11%)	1522 (21%)	999 (200 - 2398)	1199 (450 - 2199)
Don't Know/Don't want to answer	43 (12%)	893 (12%)	799 (167 - 1399)	799 (283 - 1832)
Missing	11 (3%)	159 (2%)	500 (350 - 1324)	599 (200 - 1399)
**Age Category (years) ‡**				
40 - 50	59 (16%)	2869 (39%)	799 (233 - 1898)	899 (300 - 1898)
51 - 60	164 (45%)	2966 (40%)	599 (125 - 1399)	841 (300 - 1898)
61 - 70	138 (38%)	1479 (20%)	799 (100 - 1998)	799 (300 - 1798)
>70	0 (0%)	7 (0.1%)		250 (300 - 1798)

	Respondents N (%)		Physical Activity† Median (Inter-Quartile Range)	
	**Diabetes**	**Non-Diabetes**	**Diabetes**	**Non-Diabetes**
Missing	1 (0.2%)	17 (0.2%)		799 (400- 1499)
**Neighborhood Disadvantage (Quantiles)**				
Quantile 1 (most disadvantaged)	69 (19%)	862 (12%)	599 (100 - 1798)	749 (200 - 1598)
Quantile 2	88 (24%)	1284 (17%)	699 (200 - 1748)	699 (233 - 1598)
Quantile 3	68 (19%)	1309 (18%)	599 (92 - 1411)	799 (300 - 1798)
Quantile 4	68 (19%)	1702 (23%)	749 (183 - 1523)	932 (300 - 1998)
Quantile 5 (least disadvantaged)	53 (15%)	1956 (27%)	999 (200 - 2131)	999 (400 - 2098)
Missing	16 (4%)	225 (3%)	949 (75 - 2298)	799 (300 - 1898)

† MET.minutes/week. Calculated at first wave with data available within waves 2 to 5. Not reported when <5 people.

‡ Entered as a continuous variable in the models.
